# Lung Organoids—The Ultimate Tool to Dissect Pulmonary Diseases?

**DOI:** 10.3389/fcell.2022.899368

**Published:** 2022-07-13

**Authors:** Veronika Bosáková, Marco De Zuani, Lucie Sládková, Zuzana Garlíková, Shyam Sushama Jose, Teresa Zelante, Marcela Hortová Kohoutková, Jan Frič

**Affiliations:** ^1^ International Clinical Research Center, St. Anne’s University Hospital Brno, Brno, Czechia; ^2^ Department of Biology, Faculty of Medicine, Masaryk University, Brno, Czechia; ^3^ Institute of Hematology and Blood Transfusion, Prague, Czechia; ^4^ Department of Cell Biology, Faculty of Science, Charles University, Prague, Czechia; ^5^ Department of Medicine and Surgery, University of Perugia, Perugia, Italy

**Keywords:** induced pluripotent stem cells, lung organoids, cystic fibrosis - CF, human disease modelling, 3D structure, chronic obstructive pulmonary disease, lung cancer

## Abstract

Organoids are complex multicellular three-dimensional (3D) *in vitro* models that are designed to allow accurate studies of the molecular processes and pathologies of human organs. Organoids can be derived from a variety of cell types, such as human primary progenitor cells, pluripotent stem cells, or tumor-derived cells and can be co-cultured with immune or microbial cells to further mimic the tissue niche. Here, we focus on the development of 3D lung organoids and their use as disease models and drug screening tools. We introduce the various experimental approaches used to model complex human diseases and analyze their advantages and disadvantages. We also discuss validation of the organoids and their physiological relevance to the study of lung diseases. Furthermore, we summarize the current use of lung organoids as models of host-pathogen interactions and human lung diseases such as cystic fibrosis, chronic obstructive pulmonary disease, or SARS-CoV-2 infection. Moreover, we discuss the use of lung organoids derived from tumor cells as lung cancer models and their application in personalized cancer medicine research. Finally, we outline the future of research in the field of human induced pluripotent stem cell-derived organoids.

## 1 Introduction

Respiratory disorders such as asthma, chronic obstructive pulmonary disease (COPD), and idiopathic pulmonary fibrosis (IPF) affect millions of people worldwide, accounting for approximately 8% of global mortality (Dwyer-Lindgren et al., 2017). Lung tumors and airborne infections add to the burden of lung disease-associated morbidity and mortality, with the ongoing COVID-19 pandemic demonstrating the potential impact of viral lung disease on the worldwide population (Pollard et al., 2020; Kumar et al., 2021). Despite intensive research efforts spanning decades, our knowledge of lung biology and its interaction with the disease process remains incomplete. For example, key questions remain around the nature of lung stem cells, their roles in tissue regeneration and their therapeutic potential. A better understanding of the lung stem cell progenitor’s capacity to generate the various cell types of the adult lung and unraveling the pathways involved in their responses to injury could lead to the discovery of more efficient treatments for numerous pulmonary diseases. Thus, there is a persistent and urgent need to develop more relevant *in vitro* models of the human lung that authentically recapitulate the organ’s physiology and can be used to elucidate the mechanisms and biomarkers of pulmonary disease, as well as to test and guide the development of new treatments for conditions affecting the airways.

Studies of lung biology have, until recently, been limited by the unique structural and cellular complexity of the organ. The human lung comprises over 40 different types of cells (Franks et al., 2008; Cunniff et al., 2021; Varghese et al., 2022), arranged in a complex three-dimensional (3D) architecture that is designed to withstand the continuous dynamic mechanical stress associated with respiratory movements (Nossa et al., 2021) while existing and functioning at the air-tissue-interface, in the presence of a varied and interactive microbiota (Paolicelli et al., 2019; Barcik et al., 2020). Studies of primary tissue *ex vivo* cannot provide dynamic information, while the relative inaccessibility of the lung and its constant movement have precluded the widespread use of intra-vital imaging in pre-clinical models. Together, these challenges have driven researchers to develop and explore the use of lung organoids (LOs). These 3D *in vitro* models of the lung incorporate multiple cell types derived from induced pluripotent stem cells (iPSCs) (Clevers, 2016; Paolicelli et al., 2019; Jose et al., 2020; Wang et al., 2020a) and offer the potential to answer long-standing questions about lung biology in health and disease.

In this review we outline the unique features of the lung that make studying the organ and its pathologies such a challenge, then summarize the pre-organoid era leading to the development of these latest models. We then review the different approaches used to generate LOs, and report the latest findings made in the field, before finally looking at the direction of pulmonary research in the future.

## 2 The Pre-Organoid era: Early Attempts to Model the Human Lung

While all organs are, to some degree, complex, the human lung is remarkably so. While the stomach (Busslinger et al., 2021) and liver (Ramachandran et al., 2020), for example, are comprised of four or five main cell types, the lung contains more than 40 (Franks et al., 2008; Cunniff et al., 2021; Varghese et al., 2022), varying in proportion depending on the region of the lung and exhibiting clear patterns of polarization at the air-liquid interface (Castellani et al., 2018). Thus, the development of reliable *in vitro* lung models is a challenge on a grand scale (Castellani et al., 2018).

Accordingly, for many years, studies of lung tissue either employed a reductionist approach based on 2D culture of human cells, or relied on animal models, in which the 3D structure of the lung is intact. Together with increasing use of tools such as genetic engineering and inducible gene expression, these approaches have enabled us to better understand the molecular basis of some lung diseases (Guilbault et al., 2007; Williams and Roman, 2016; Lambrecht et al., 2019). However, 2D cultures of human cells have clear drawbacks for the study of an organ with a function that is tightly linked to its 3D organization and multiple studies have shown that the overall ability of rodent models in particular to accurately replicate human pulmonary diseases is limited (Shmidt and Nitkin, 2004; Matute-Bello et al., 2008; Wang et al., 2008). Thus, there is mounting evidence for the need to develop human cell-based systems to study pulmonary disease.

### 2.1 Basic 2D Cultures of Human Lung Cells

Prior to the advent of 3D tissue culture methods, many studies of the lung used human primary cells and/or cell lines derived from healthy pulmonary or tumor tissues, grown in monolayer cultures (Gottschling et al., 2012). The 2D models are ideal for high-throughput drug screening and their generation from cell lines with histological and genetic changes allows modeling of different clinical conditions and drug responses (Huang et al., 2021). Gazdar et al. (2010) reviewed the important discoveries made using 2D models such as, the spectrum of TP53 gene mutations in lung cancer (Takahashi et al., 1989), mechanisms of resistance in EGFR mutant cells (Kwak et al., 2005; Pao et al., 2005; Engelman et al., 2007) and the description of BRAF mutations in various tumors, including lung (Davies et al., 2002; Pratilas et al., 2008).

While these models have the advantage of human origin, they are constrained by the culture’s limited amount of extracellular matrix and the absence of different lung cell subtypes—including progenitors—and their inability to replicate the morphology or structural features of the *in vivo* lung architecture. The significance of these factors has become even more apparent in recent years: for example, we now know that lung-infiltrating immune cells are fundamental to the biology of several lung diseases, including COPD (Huang et al., 2019; David et al., 2021), IPF (Tanabe et al., 2020; van Geffen et al., 2021; Zhu et al., 2021), and non-small cell lung cancer (Tamminga et al., 2020). Furthermore, recent studies have illustrated the central role of tissue polarity in lung conditions, which can alter the functions of native cells in terms of spreading, migrating, and sensing soluble factors and other ligands (Duval et al., 2017). This implies limited relevance of findings from 2D cell cultures or isolated primary cells.

### 2.2 Rodent Models of Human Pulmonary Disorders

**FIGURE 1:**
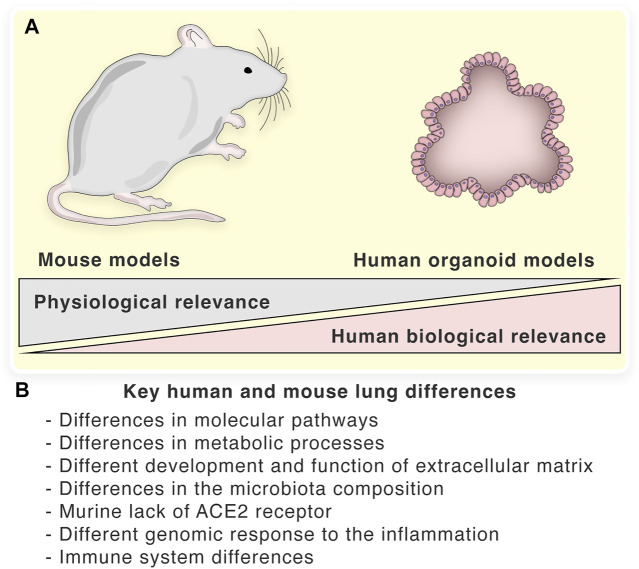
Key human and mouse lung differences: **(A)**—Biological and physiological relevance of mouse and organoid models. **(B)**—The main differences between human and mouse lungs.

Although the etiology of human lung diseases is complicated and multi-factorial, there are three broad underlying forces: genetic mutation/variation/predisposition, environmental exposure/influences, and infection by a pathogen. Animal models have been used widely to investigate the mechanisms of each of these forces. While such studies have generated important insights into human lung disorders, there is a growing body of evidence that indicates their lack of ability to achieve progress in the field. Molecular characterization of human lungs using proteomic, transcriptomic, and *in silico* approaches, such as the Molecular Atlas of Lung Development Program (LungMAP), revealed major molecular differences between mouse and human lung epithelial tissues (Ardini-Poleske et al., 2017; Pan et al., 2019). Recent studies also showed that human lungs significantly differ from those of mouse models in several key metabolic processes as well as molecular pathways regulating the development and function of the lung extracellular matrix (Wang et al., 2015; Aichler et al., 2018). The main differences between human and mouse lungs are summarized in Figure 1. Below, we note key examples of the types of animal models commonly in use, acknowledging the advances and also noting their limitations that are driving the design of ever better LOs.

#### 2.2.1 Models of Genetic Diseases Affecting the Lungs

CF is among the most common autosomal recessive diseases in humans and is caused by mutation of the cystic fibrosis transmembrane conductance regulator (*CFTR*) gene. Genetically modified rodents bearing one or several of the more than 2,000 *CFTR* mutations that can result in CF in humans (De Boeck, 2020) have been used to model the condition, as reviewed by Semaniakou et al. (2018). However, although these models bear mutations known to be associated with the onset of CF in humans, the disease phenotypes in these mice vary in severity and pathology. These differences have been attributed to the rodent’s lack of specific cell types, receptors, and mediators involved in CF (Grubb and Boucher, 1999; Pan et al., 2019). Recent studies indicate that the differences in the microbial composition of the murine and human lung could also be a key factor (Semaniakou et al., 2018). Taken together, the distinct microbiota, alongside the molecular and physiological differences between mouse and human lungs, mean that murine models of CF are limited in their ability to advance our knowledge.

The same pattern emerges for the study of IPF—a chronic progressive disease characterized by scarring of the lung tissue—which has incompletely understood environmental and genetic components to its etiology (Chanda et al., 2019). The main mouse model of IPF involves the induction of pulmonary fibrosis by direct administration of bleomycin into the lungs of rodents (Walters and Kleeberger, 2008), with more recent developments including transgenic overexpression of pro-fibrotic cytokines such as TGF-β, TNF-α, IL-1β, and IL-13 in mice (Tashiro et al., 2017). However, despite showing fibrotic features, mouse models of IPF often display heterogeneous disease kinetics, especially in terms of the severity and extent of pulmonary lesions and levels of different cytokines (Moeller et al., 2008). Humanized mice have also been used as recipients of injected IPF patient lung biopsy material or tissue explants, which has allowed researchers to study the biology of the resulting lung remodeling and fibrotic lung phenotype (Habiel et al., 2018). As a result of murine studies, two IPF drugs have been developed, pirfenidone (King et al., 2014) and nintedanib (Richeldi et al., 2014), but neither are able to cure the disease and patient survival remains poor, with a median lifespan of 3.8 years post-diagnosis (Raghu et al., 2014).

#### 2.2.2 Models of Pulmonary Infections

Another challenge is the development of relevant preclinical models of human pulmonary infections. Rodents and humans differ in their susceptibility to many pulmonary pathogens. This is particularly clear in mouse models of lung conditions that, in humans, develop through recurrent exacerbations of pulmonary infections including those by *Staphylococcus aureus*, *Haemophilus influenzae, Pseudomonas aeruginosa*, and *Burkholderia cepacian* (Semaniakou et al., 2018). Again, the difference in the microbes colonizing laboratory mice and humans is likely a major factor limiting the applicability of these models, and it is not yet clear whether this challenge can be overcome, although some attempts are being made [reviewed in Chang *et al.* (Chang and Sharma, 2020)]. Human viral lung infections are also difficult to study in murine models. For instance, investigations of human infection with the pathogenic avian influenza virus H5N1 in these models is impeded by the absence of the epithelial receptor for viral entry in mice (Ibricevic et al., 2006). Similarly, mice lack the ACE2 receptor required for severe acute respiratory syndrome coronavirus 2 (SARS-CoV-2) infection, initially dramatically limiting their applicability in this field. Although the development of a mouse model expressing the human ACE2 receptor was recently reported (Cleary et al., 2020), it is still unclear whether the response of mice to the virus mimics that of humans, and whether they express other as yet unidentified co-factors involved in infection/pathology.

Sepsis is a condition characterized by life-threatening organ dysfunction, and is caused by the host response to infection, most commonly by bacteria (Minasyan, 2019; Reyes et al., 2020) but also by some viruses (Singer et al., 2016; Hortova-Kohoutkova et al., 2020; Hortova-Kohoutkova et al., 2021). In the respiratory system, sepsis manifests clinically as acute lung injury (ALI) or, the more severe acute respiratory distress syndrome (ARDS) (Sevransky et al., 2009; Angus and van der Poll, 2013). Sepsis-induced ALI is marked by a “cytokine storm” that triggers inflammation and is followed by uncontrolled infiltration of pulmonary tissue by neutrophils (Aziz et al., 2013), while ARDS also involves pulmonary edema caused by alveolar injury that results in dangerously low oxygen levels in the blood (Sweeney and McAuley, 2016). Respiratory-infection-linked sepsis deaths totaled 1.8 million globally in 2017 (Rudd et al., 2020) driving urgent research into the condition. Sepsis has been experimentally induced in rodents by the administration of microbial components or living pathogens (e.g., *Escherichia coli*, or *S. aureus*) into the bloodstream, peritoneal cavity, or trachea (Karzai et al., 2003; Wang et al., 2004; Charavaryamath et al., 2006; Stahl et al., 2013; Kapicibasi et al., 2020). These models have shown that sepsis induced in mice by injection of peptidoglycan causes organ injury, organ, and systemic inflammation (Wang et al., 2004). Similarly, injection of a high dose of LPS and alphatoxin induces a systemic inflammatory response syndrome in rodents (Stahl et al., 2013). However, despite extensive animal studies, clinical translation has been largely unsuccessful. In this case, one of the reasons for the poor translation of the mouse data to humans is likely due to their markedly different genomic response to inflammation. A study comparing mouse and human endotoxemia showed no correlation between the top 10 most downregulated signaling pathways in the two species. In contrast to mouse models, the response to injury in humans was dominated by upregulation of genes related to innate immune response and downregulation of pathways related to adaptive immunity (Seok et al., 2013). Therefore, one must conclude that the balance of harm to the animal—in this case significant suffering is induced during sepsis modeling—and the low potential for valuable clinical insight, renders the use of such models highly undesirable for future research (Nandi et al., 2020).

#### 2.2.3 Animal Models of Human Lung Cancer

Since the first animal model of leukemia was reported in 1950, many types of animal models of cancer have been developed to understand tumor biology and test drug efficacy. One of the simplest human tumor models is created by injecting tumor cell lines into immunodeficient mice. However, the pressure to develop more standardized model emerged due to unanticipated and unpredictable phenotype alterations compared to the original tumor. The patient-derived xenograft (PDX) model represents a reliable translation research tool that accurately mimics parental tumor tissue. It is generated by implanting a small tissue sample into highly immunodeficient mice. The great advantages of PDX models are in preserving the histologic features, genetic aberrations and gene expression profiles of the original tumor, as well as matching the characteristics of inflammation and the responses to chemotherapeutics and other anti-tumor drugs of the parental tissue. Therefore, PDX models are a valuable tool in tumor biology research, development of novel anti-cancer therapeutics, and pre-clinical drug screening (Lai et al., 2017; Bleijs et al., 2019; Yoshida, 2020). Although PDX models represent highly complex systems that accurately replicate tumor tissue *in vivo*, they also have several drawbacks, for example, the low success rate (30%–40%, lack of efficacy and the high cost of their establishment in terms of time and money, as well as their limited potential for application in high-throughput studies (Kim et al., 2019; Li et al., 2020). These features render PDX models unsuitable for use in the personalized treatment industry and other models suitable for high-throughput research are urgently required.

In lung cancer modeling, PDXs were reported in development of a novel anti-tumor therapeutic approaches, for example, in evaluation of microwave hyperthermia therapy (Motomura et al., 2010). Furthermore, PDX models of lung cancer have been used in pre-clinical drug testing (Hai et al., 2020; Li et al., 2020; Shi et al., 2020; Wang et al., 2020b), which is discussed in the following sections.

## 3 The Birth of Human Lung Organoids: Physiologically Relevant Models of Pulmonary Tissues

To overcome the limitations of classical 2D cultures and inter-species models, the use of lung tissue organoids has been pioneered in the field of lung research. Organoids are 3D “organ in a dish” models that aim to recreate key aspects of the *in vivo* structure of tissues using a mixture/variety of cell types from the species of interest to generate a relevant microenvironment in which cells within the organoid exhibit key aspects of the function of that organ (Lancaster and Huch, 2019).

Research into organoids was initially pioneered in the field of cancer studies, with cultured 3D structures based on simple air-medium interface system used to characterize the histological features of tumors (Kondo and Inoue, 2019). A significant advance in our ability to model non-malignant cells was the innovative demonstration by Sato et al. (2009) that individual Lgr5^+^ stem cells isolated from small intestinal crypts of adult mice could be grown and maintained in 3D cultures in which they regenerated the intestinal niche, comprising villus-like structures and fully differentiated epithelial cells. Since then, the use of tissue organoids in disease modeling research has expanded rapidly, with more than 3,000 scientific articles now published in this field alone (Lancaster and Huch, 2019).

It is now possible to generate LOs from cultures of pluripotent stem cells (PSCs) (including embryonic stem cells), induced PSCs (iPSCs), or adult stem cells (ASCs). Regardless of the source cell type, a key procedure common across all protocols for organoid derivation is the embedding of cells in extracellular matrix, which serves as the basal lamina for tissue culture and supports the development of the 3D architecture (Schutgens and Clevers, 2020). Fully differentiated organoids can then be passaged, further expanded, and used for basic tissue research (Tian et al., 2021), cell interaction studies (Nikolic and Rawlins, 2017), and cancer drug testing (Clevers, 2016; Drost and Clevers, 2018; Kim et al., 2019).

### 3.1 Different Methods for Generating Lung Organoids

As mentioned above, human LOs can now be derived through several routes and different biological materials, each with its own strengths and limitations. Selecting the most appropriate method for each application is of key importance, but with careful application of the methodologies mentioned below, valuable insights into lung biology and disease pathology can be made.

#### 3.1.1 Generation of Primary Lung Progenitor Cell-Derived Organoids

Early protocols for LO differentiation were based on the use of primary lung progenitor cells (Leeman et al., 2019; Rabata et al., 2020; Schutgens and Clevers, 2020), or transformed lung cancer cells (Neal and Kuo, 2016; Sato et al., 2017). Several methods have been published describing the isolation of these cells from lung tissue that was first subjected to mechanical disruption followed by digestion using enzymes (Katsura et al., 2020; Salahudeen et al., 2020; Lamers et al., 2021; Tindle et al., 2021). Once a single-cell suspension has been generated, the cells are either directly seeded into Matrigel droplets for organoid culture or sorted based on the expression of cell-type-specific markers for organoid cultures that require a more homogeneous population of a particular cell type (Katsura et al., 2020).

Importantly, it is also possible to generate LOs from specific lung regions to model their physiology more accurately. For example, human lung basal cells can be isolated from either the trachea or upper bronchia, after which they are expanded *ex vivo* and stem cells are isolated for embedding into the matrix (Schutgens and Clevers, 2020). Similarly, alveolar-like organoids can be generated by sorting of type II alveolar cells identified as CD45^−^CD31^−^Epcam^+^HTII^+^ cells from dissociated lung tissue dissociation and mixing them with MRC5 fibroblasts before embedding in Matrigel (Barkauskas et al., 2013). A second method of alveolar organoid generation involves co-culture of epithelial progenitors with stromal fibroblasts (Tan et al., 2017), although this approach is less commonly used. More advanced LOs exhibiting a limited lung-like structure have been generated by mixing epithelial and fibroblastic cells using multi-layered microfluidic devices that enable the formation of differentiated LOs with morphological and secretory cellular phenotypes similar to those in human lungs (Hegab et al., 2015; Gkatzis et al., 2018). Similarly, organoids derived from alveolar or small airway epithelial cells have been co-cultured with mesenchymal stromal cells (MSCs) to promote alveolar organoid formation (Leeman et al., 2019). Leeman et al. (2019) also showed that ASC-derived LOs exhibit increased alveolar differentiation and decreased self-renewal when cultured with MSC-conditioned medium, thus demonstrating that MSC-secreted factors are important for the self-organization of lung epithelial organoids. Despite the achievements in the field of primary lung progenitor cell-derived organoid research, some limitations of these *in vitro* models remain. First, primary cell-derived LOs lack the mesenchymal cells that produce factors to support LO growth and differentiation (Leeman et al., 2019). Therefore, the successful generation of LOs requires either co-cultivation with mesenchymal cells or supplementation of the medium with these factors. Furthermore, cell-derived organoids are formed from only a limited number of cell types, which does not accurately mirror the complex structures of the human lung. Finally, primary cell-derived organoids require lung tissue biopsies, which provide only a limited amount of material for LO differentiation.

#### 3.1.2 Generation of Induced Pluripotent Stem Cells-Derived Lung Organoids

**FIGURE 2:**
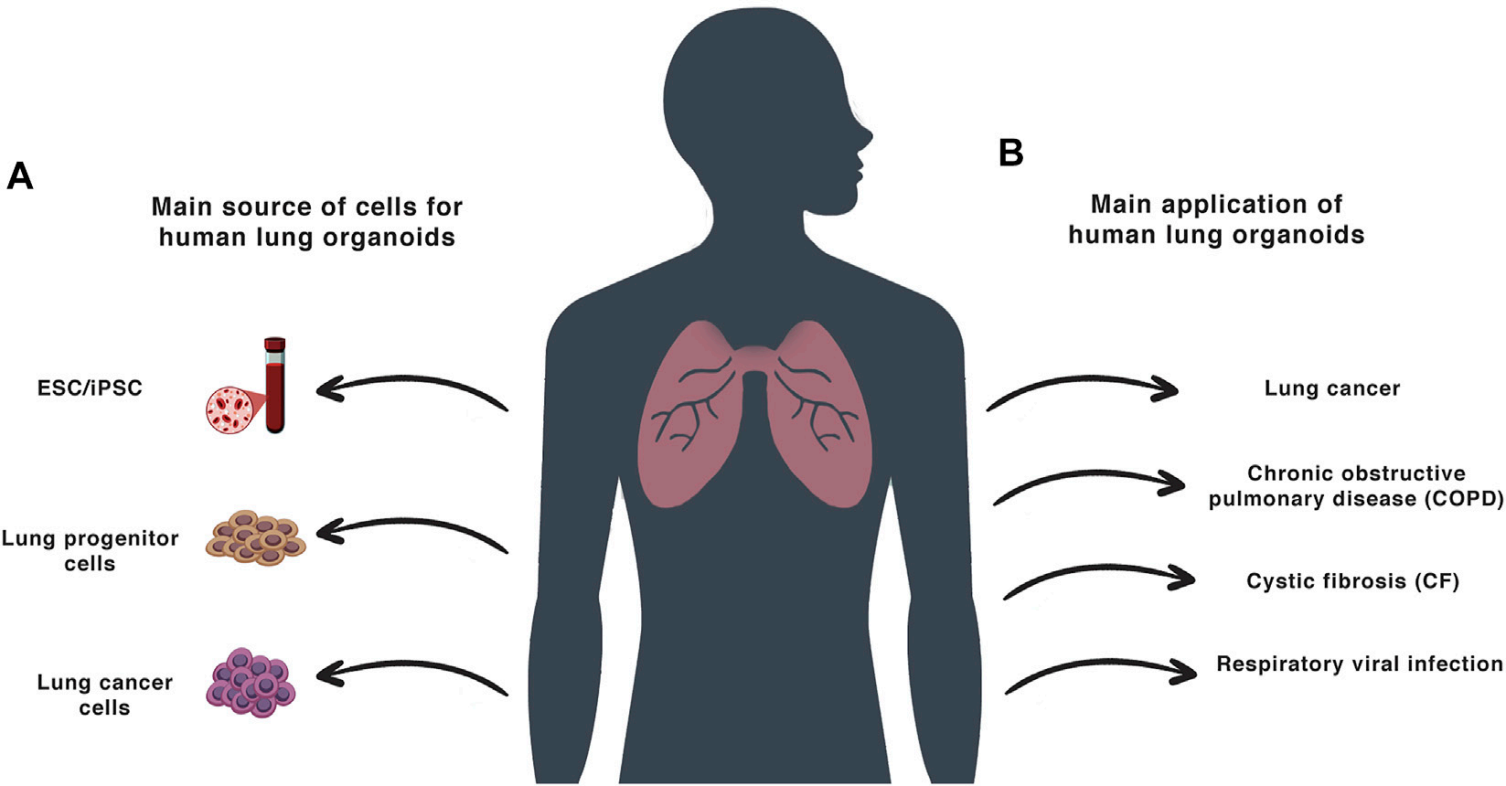
Lung organoids: **(A)**—The main sources for the creation of the human LOs. **(B)**—The main application of human LO models.

iPSC-based organoids can be generated by reprogramming somatic cells from patients (often easily accessible skin fibroblasts or peripheral blood mononuclear cells), making them a uniquely valuable and versatile tool for clinical research (Figure 2). This approach has revolutionized the study of genetic disorders in particular, superseding the previous ASC-based organoids, that were limited by their lack of MSCs and fibroblastic stromal cells, which are required for tissue-like structures and functions (Schutgens and Clevers, 2020). Thus, the use of PSCs to generate LOs with both epithelial and mesenchymal components has been a breakthrough in the field, and these organoids are now being utilized to study lung development and lung diseases across a broad range of fields (Choi et al., 2016).

**FIGURE 3:**
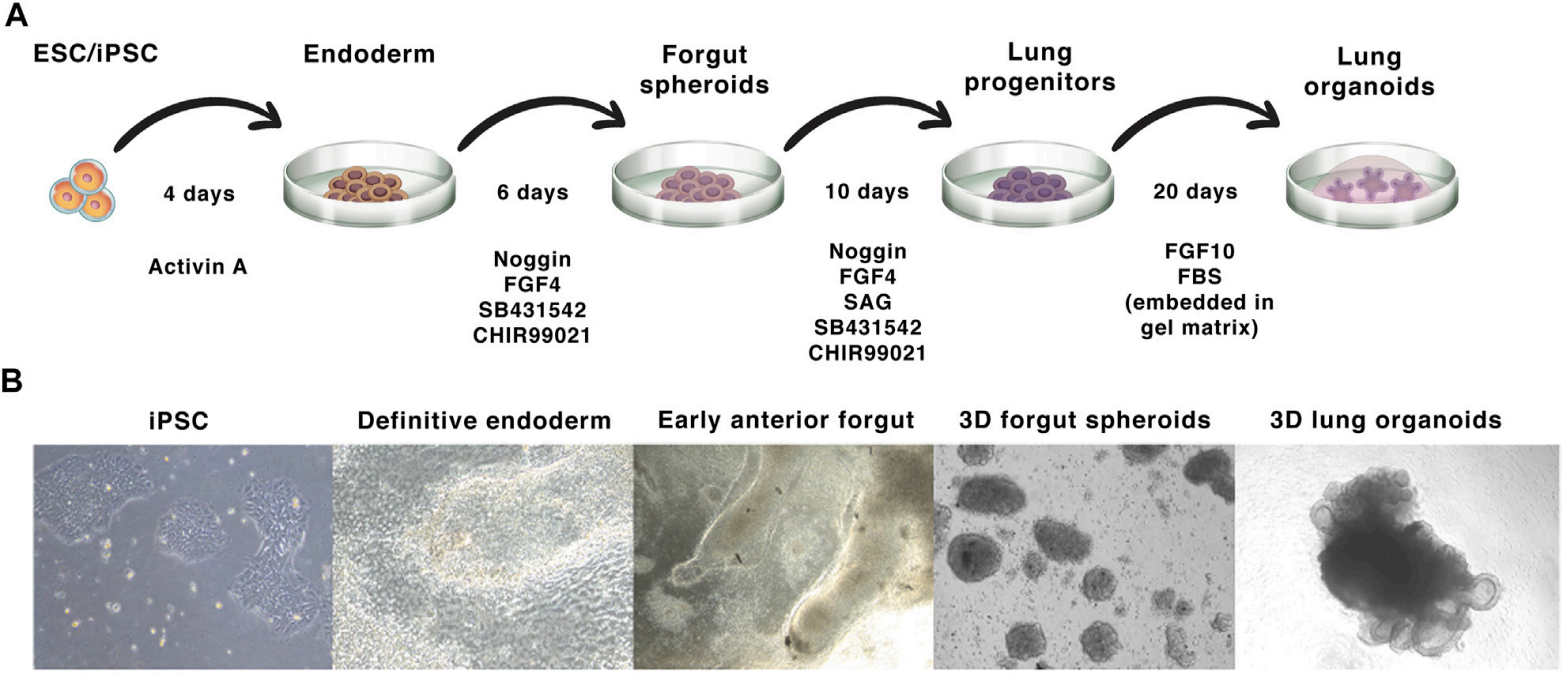
iPSC-derived lung organoid differentiation: **(A)**—The protocol starts with the induction of iPSC colonies to form the definitive endoderm, which is then differentiated into foregut spheroids. These spheroids are then embedded in a gel basal matrix and further differentiated into LOs. **(B)**—Bright-field images of the different stages of LO development from iPSCs.

**FIGURE 4:**
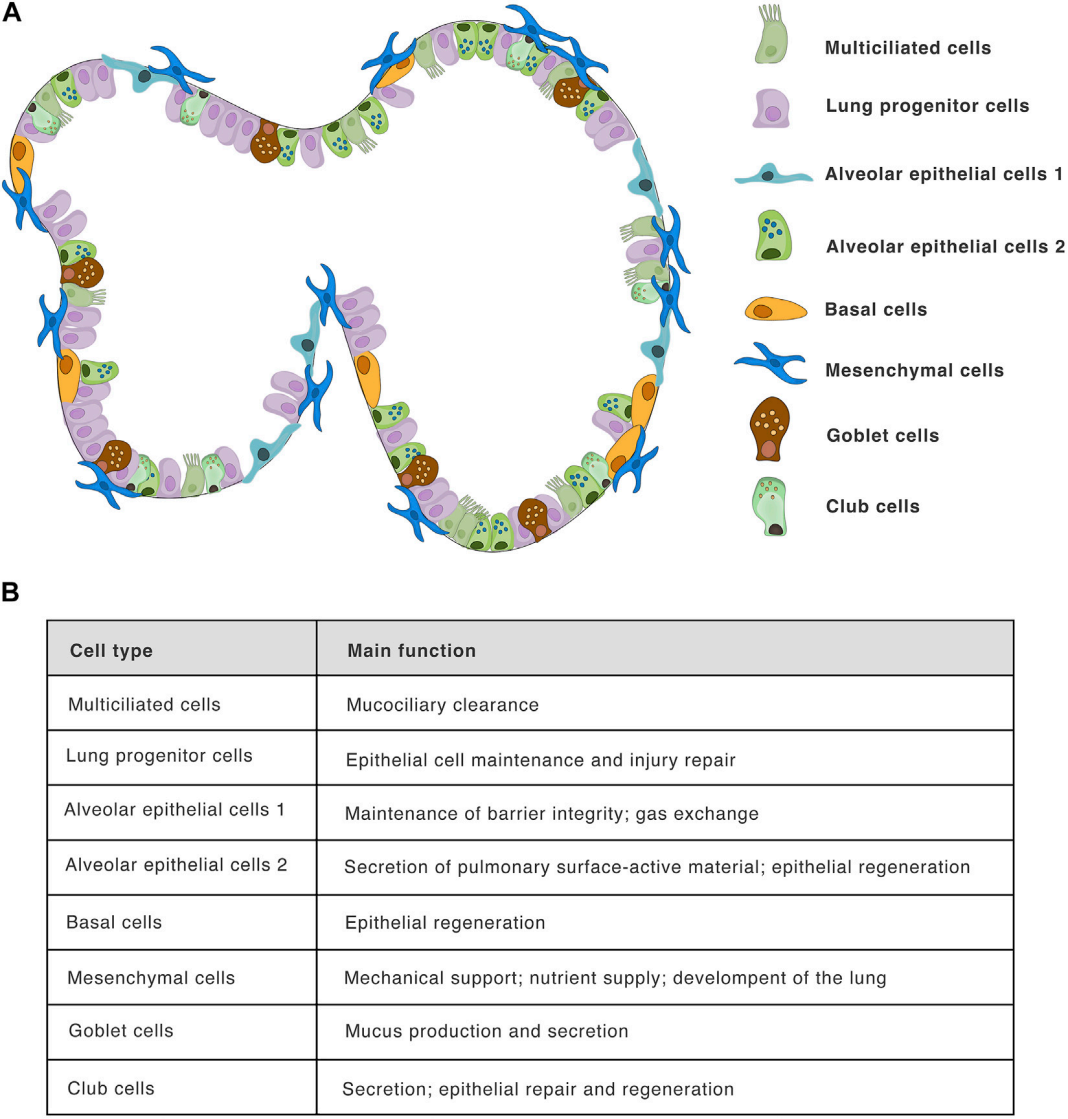
Lung iPSC-derived organoid composition: **(A)**—The structure of human LOs and their cellular composition. The scheme shows selected major cell types forming in published iPSC-derived LOs models, including club cells, goblet cells, basal cells, alveolar epithelial cells 1 and 2, multiciliated epithelial cells, lung progenitor cells, and mesenchymal cells. While human lung tissue comprises more than 40 different cell type, including infiltrating immune cells, the iPSC-derived organoids models provide unique opportunity to study the “tissue” without the infiltrating hematopoietic cells. Although, iPSC-derived organoids represent excellent *in vitro* model, they do not fully mimic the human lung tissue complexity. **(B)**—Main function of mentioned cell types in human the lung.

The process of LO differentiation follows that of normal embryogenesis, in which fetal lungs arise from the anterior foregut endoderm, forming the bronchial, and alveolar tissues (Schittny, 2017). *In vitro*, this process is driven/directed by sequential activation and inhibition of different signaling pathways using growth factors and small molecules. The iPSC-derived LO differentiation protocol was firstly described by Dye et al. (2015). This protocol involves the initial differentiation of either ESCs or iPSCs into definitive endoderm by treatment with activin A for a period of 4 days (Figure 3, days 1–4). This endodermal layer is then differentiated to form anterior foregut spheroids by using a combination of noggin, the TGFβ/activin/NODAL pathway inhibitor SB431542, FGF4, and the GSK3 inhibitor CHIR99021 (Figure 3, days 4–10). These signals are sufficient to induce both mesenchymal and epithelial cell populations within the anterior foregut spheroids. The activation of the WNT and FGF pathway and the simultaneous inhibition of the BMP/TGFβ signalling in PSCs was found to be optimal for the generation of lung tissue expressing NKX2.1^+^, a key transcription factor that regulates pulmonary development (Minoo et al., 1995; Choi et al., 2016). For the generation of NKX2.1^+^ E-cadherin^+^ foregut spheroids, the sonic hedgehog activator SAG is also added on days 4–10. Finally, NKX2.1^+^ SOX9^+^ spheroids are embedded into a gel matrix and expanded in an FGF10-containing medium, leading to the development of 3D LOs comprising both P63^+^ basal cells and FOXJ1^+^ ciliated epithelial cells (Dye et al., 2015; De Luca et al., 2017). Importantly, these organoids also exhibit tissue polarity, evidenced by the localization of acetyl-tubulin and E-cadherin markers (Jose et al., 2020). As previously mentioned, human lung tissue comprises more then 40 cell types (Franks et al., 2008; Cunniff et al., 2021; Varghese et al., 2022), including infiltrating immune cells. However, iPSC-derived organoids do not consist of any cells of hematopoietic origin, which can be an experimental advantage allowing controlled cocultures, such as Jose et al. (2020) showed for monocytes. Moreover, distinct protocols differ in the cell types composition within the organoids. The composition of several cell types forming iPSC-derived organoids is shown in Figure 4, the overall complexity of iPSCs model indeed does not reach completely the normal lungs.

A recent advance in LO research is represented by reporter iPSCs, which allows the differentiation of different cell types to be tracked within the organoid. Pioneered by Sharma et al. (2018), this method involves fluorescent tagging of endogenous proteins in a stable human iPSC line using CRISPR/Cas9 genome editing technology (Sharma et al., 2018). Since almost any intracellular protein can be tagged, this system represents a powerful tool with the potential for application in many fields of research. Notably, this technique has recently been used in our own laboratory to study the activation of transcriptional factors such as NF-κB during LO differentiation and stimulation (Jose et al., 2020). Hawkins et al. (2021) published the protocol for directed differentiation of human iPSCs into airway basal cells allowing use of the iPSC NKX2-1^GFP^/TP63^tdTomato^ dual reporter system. This protocol facilitates tracking of the differentiation state of the cells and sorting of the positive population to obtain pure culture of lung progenitor cells (Hawkins et al., 2021). In another study, the fluorescent reporter NKX2-1 and SFTPC iPSCs line were used to generate and purify alveolar epithelial type 2 cells (AEC2s) (Jacob et al., 2017). Importantly, this approach provides opportunities to study AEC dysfunction, which has been implicated in the pathogenesis of many lung disorders (Hawkins et al., 2021).

Advances in iPSC-derived LO generation have led to the generation of organoids with close transcriptional similarities to human fetal lung (Dye et al., 2016); however, their ability to recapitulate the characteristics of the adult tissue remains to be fully defined.

## 4 The Application of Lung Organoids

The special characteristics of organoids make them an excellent model for a wide range of basic and translational investigations, including drug testing, genetic screening, and disease modeling (Table 1). The major advantage of LO cultures is the interdependent existence of epithelial and mesenchymal cell populations, which—alongside epithelial polarization—is required for proper pulmonary function *in vivo* (Tan et al., 2017).

**TABLE 1:** Lung organoids used for disease investigation: Disease studies where 3D LOs were used as experimental models.

Disease modeled	Source cell type	References
Lung cancer	Human primary tumor tissue/cells	Jung et al. (2019); Kim et al. (2019)
	Patient-derived xenograft models	Shi et al. (2020)
	Human lung cancer cell lines	Ramamoorthy et al. (2019)
	Human ESCs	Chen et al. (2017)
COPD	Primary human lung epithelial cells	Ng-Blichfeldt et al. (2018)
	Human lung epithelial cell lines	Tan et al. (2017)
CF	Patient-derived iPSCs	Mou et al. (2012); Wong et al. (2012); Firth et al. (2015)
	Lung cell pellet from the broncho‐alveolar lavage fluid of patients	Sachs et al. (2019)
IPF	Patient-derived iPSCs	Wilkinson et al. (2017); Strikoudis et al. (2019)
	Human ESCs	Chen et al. (2017)
Neonatal respiratory distress syndrome	Patient-derived iPSCs	Jacob et al. (2017)
Interstitial lung disease	Patient-derived iPSCs	Leibel et al. (2019)
Bronchopulmonary dysplasia	Human fetal lung fibroblasts	Sucre et al. (2016)
Pulmonary metaplasia	Normal primary human epithelial cells	Danahay et al. (2015)
Pulmonary edema	Human pulmonary epithelial cells and microvascular endothelial cells used to form 3D-lung organoids on a chip	Huh et al. (2012)
Lung inflammation	Human 3D differentiated airway epithelium cultured on-chip (inflammation induced by IL-13)	Benam et al. (2016)
	Mouse lung tissue (inflammation induced by bacterial flagellar hooks stimulation)	Shen et al. (2017)
	Mouse type 2 alveolar epithelial cells (inflammation induced by IL-1β and TNFα)	Katsura et al. (2019)
Lung tissue injury and regeneration	Primary mouse lung epithelial cells, endothelial cells, and MSCs	Leeman et al. (2019); Riemondy et al. (2019)
Respiratory viral infection	*IRF7* mutant patient-derived iPSCs (influenza virus infection)	Ciancanelli et al. (2015)
	Mouse epithelial stem/progenitor cells (influenza virus infection)	Quantius et al. (2016)
	Human ESCs (respiratory syncytial virus infection)	Chen et al. (2017)
	Human ESCs (parainfluenza virus infection)	Porotto et al. (2019)
	Human airway epithelial cell cultures (parechovirus infection)	Karelehto et al. (2018)
	Human alveolar epithelial type II of KRT5+ basal cells (severe acute respiratory syndrome coronavirus 2)	Salahudeen et al. (2020)
	Human alveolar type 2 cells/pneumocytes (severe acute respiratory syndrome coronavirus 2)	Katsura et al. (2020)
	Human epithelial progenitor cells (severe acute respiratory syndrome coronavirus 2)	Xu et al. (2021)
	Human alveolar type 2 cells (severe acute respiratory syndrome coronavirus 2)	Ebisudani et al. (2021)
	Primary human lung tissue (enterovirus infection)	van der Sanden et al. (2018)

Used abbreviations: COPD, chronic obstructive pulmonary disease; CF, cystic fibrosis; IPF, idiopathic pulmonary fibrosis; ESCs, embryonal stem cells; iPSCs, induced pluripotent stem cells; MSCs, mesenchymal stromal cells.

### 4.1 Lung Organoids as Models of Genetic Diseases Affecting the Lungs

LOs have been used successfully to investigate the pathogenesis of CF and protocols have been published for generating disease-specific lung progenitor cells from human CF patient-derived iPSCs (Mou et al., 2012; Wong et al., 2012). The value of this approach lies in the use of a human model, as the murine models may not be exact phenocopy of the human lung disease (Clarke et al., 1992; Snouwaert et al., 1992; Guilbault et al., 2007; Mou et al., 2012). Moreover, patient-derived iPSCs can be used to study the clinical variability of CF without the need for prior analysis of the genetic background of patients (Mou et al., 2012). Furthermore, Mou et al. (2012) used modified RNAs as a replacement for viral vectors to establish iPSC lines that are not genetically modified and therefore, provide an advantage for potential clinical use. Wong et al. (2012) validated the applicability of iPSC-derived epithelial cells as an *in vitro* model for the drug screening. In this study, CF patient-specific iPSC-derived lung epithelial cells were used to test a novel small molecule compound called a “corrector”, which is able to restore the trafficking of a mutant CFTR protein to the plasma membrane. “Corrector” treatment of cells with the F508del CF mutation resulted in the enhanced plasma membrane localization of the CFTR protein (Wong et al., 2012). Finally, this approach provides the ability to test drugs for personalized medicine and the possible future development of regenerative medicine for lung disorders. An additional advantage of iPSC-derived LOs in the study of lung fibrosis lies in their capacity for expansion of lung stem cell populations and the induction of differentiated cells from very limited amounts of starting material. In one study, Firth et al. (2015) took advantage of this feature to generate iPSC-derived LOs from CF patient fibroblasts. These organoids exhibited fibrotic characteristics *in vitro*, which were abrogated by CRISPR-mediated CFTR correction, ultimately giving rise to normal LOs (Firth et al., 2015). Alongside these disease-modelling successes, LOs have been instrumental in advances in CF drug testing (Kim et al., 2021). In one study, fibrotic response and collagen accumulation were ameliorated in a LO model of CF by treatment with NP-011, a novel potential anti-fibrotic drug (Kim et al., 2021). In the future, the use of patient-derived organoids may hold the potential for improved diagnostics (Dekkers et al., 2013), with the hope of achieving greater insight into disease processes, and perhaps even personalized therapies for CF (Berkers et al., 2019).

The limitations of mouse models of IPF also make LOs the natural choice for advancing knowledge in the field. This is especially so given the important role of altered extracellular matrix (ECM) composition in IPF pathology, which is readily studied in organoid models of the IPF lung (Kim et al., 2021; Suezawa et al., 2021). These models have yielded important insights, including the identification of new inhibitors of fibrinogenesis, such as NP-011, which was shown to ameliorate fibrosis induced by TGFβ (Kim et al., 2021). Moreover, similar results were obtained using in a mouse model of pulmonary fibrosis, suggesting the important potential of LOs for respiratory disease modeling and drug testing (Kim et al., 2021). Another condition extensively studied using LOs is fibrotic lung injury and the associated repair mechanisms. For instance, Chen et al. (2017) recapitulated fibrotic lung disease *in vitro* in normal iPSC line-derived LOs by the introduction of a mutation in the *HPS1* gene. ECM and MSCs accumulated in affected LOs, in processes similar to those observed in *HPS1* mutant-driven lung fibrosis *in vivo* (Chen et al., 2017).

Cytokine stimulation of organoids has been investigated in the field of pulmonary research. IL-1β is produced by lung interstitial macrophages in mouse lungs following bleomycin-induced injury (Choi et al., 2020). In the alveolar region, IL-1β induces differentiation of AT2 cells into damage-associated transition progenitors (DAPTs), which further differentiate into AT1 and AT2 cells to regenerate the alveolar compartment (Choi et al., 2020). This also occurs in IL-1β-stimulated mouse AT2 organoids (Choi et al., 2020), indicating the suitability of cytokine treatment of organoids for the study of inflammation and regeneration after injury. Choi et al. (2020) also demonstrated that chronic inflammation can be mimicked by IL-1β treatment of LOs, with sustained stimulation with IL-1β found to impair the terminal differentiation of AT1 cells and cause accumulation of DAPTs. Such an increase in the number of cells with expression profiles similar to DAPTs also occurs in the lung tissue of patients with IPF (Choi et al., 2020). Interestingly, withdrawing IL-1β after 14 days of LO stimulation resulted in increased terminal differentiation of AT1 cells (Choi et al., 2020), suggesting the potential benefits of therapies targeting IL-1β in the treatment of IPF. These examples of organoid models of immune responses in various tissues indicate a promising trajectory for basic and preclinical immunological research.

### 4.2 Lung Organoids to Model Lung Infections—The Model of SARS-CoV-2 Infection

The inflammatory response plays a crucial role in pathology as well as in the regeneration of lung tissue (Lechner et al., 2017). For example, cytokine storm and cytokine-release syndrome are life-threatening systemic inflammatory events involving elevated levels of circulating cytokines and immune cell hyperactivation (Fajgenbaum and June, 2020). In experimental settings, the induction of cytokine storm-like environments can be achieved by infection of lung tissue with a pathogenic agent or by direct stimulation with relevant cytokines. The cytokine approach has the advantage of mimicking the inflammatory niches in culture, even in relatively simplified *in vitro* tissue models, which lack the complete milieu of naturally occurring cell types.

Airway epithelial cells serve as a first line of defense against pathogen attack or inflammatory stimuli. Accordingly, LOs have been exploited to help understand how epithelial cell function/dysfunction contributes to the pathogenesis of various inflammatory lung diseases and infections (Gkatzis et al., 2018).

Several organoid-based *in vitro* models of lung infection have been established, which have provided valuable insights into the underlying host-pathogen interactions at the cellular and molecular levels. The recent SARS-CoV-2 pandemic has increased the demand for, and focus on, *in vitro* models of human lung tissue to facilitate disease pathology investigations and drug testing experiments. A seminal work in the field of LO infection models was published by Chen et al. (2017), who presented a protocol to generate PSC-derived lung bud organoids consisting of mesodermal (Vim^+^, CD90^+^) and pulmonary endodermal (FOXA2^+^, NKX2.1^+^, EPCAM^+^, SOX9^+^) cells. These organoids were shown to undergo branch morphogenesis when cultured in 3D Matrigel or transplanted into a mouse, rendering the model highly relevant as branching morphogenesis is a critical step in lung tissue development (Schittny, 2017). The researchers also infected the organoids with respiratory syncytial virus (RSV), which causes small airway obstruction and bronchiolitis in infants. They revealed a process of shedding of swollen, infected cells similar to that seen in infected human lungs (Chen et al., 2017). Therefore, RSV-infected LOs represent a useful model for RSV infection research, especially as most commonly used mouse models are limited by crucial differences between human and murine physiology, especially in metabolism, which may also be due to the fact that model organisms develop faster than humans (Kim et al., 2020).

A system for the culture of organoids derived from a single adult human alveolar epithelial type II cell (AT2) has also been developed. The resulting organoids successfully supported the differentiation of AT2 cells into AT1 cells (Salahudeen et al., 2020), which together form the human lung epithelium, in which AT2 cells produce pulmonary surfactant proteins, and AT1 cells cover most of the surface area of the alveoli and perform the function of gas exchange (Cunniff et al., 2021). These organoids have also been used to model human SARS-CoV-2 infection (Salahudeen et al., 2020). Around 10% of AT2-derived organoids (specifically the SFPTC^+^ cells) and 10% of basal cell-derived organoids (specifically SCGB1A1^+^ club cells) were infected with SARS-CoV-2. These results indicate that AT2 cells are directly infected by SARS-CoV-2 and that club cells are a distal lung target population (Salahudeen et al., 2020).

Xu et al. (2021) used LOs to examine the innate cellular immune response of lung tissue during SARS-CoV-2 infection. They found that expression of receptor interacting serine/threonine-protein kinase 1 (RIPK1), an important mediator of inflammation and cell death (Mifflin et al., 2020), is upregulated in patients with COVID-19 who experience a cytokine storm, and is also activated in SARS-CoV-2-infected primary LOs. Interestingly, treating infected LOs with the RIPK1-inhibitor Nec-1s reduced the transcription of proinflammatory cytokines, as well as ACE2 and the epidermal growth factor receptor (EGFR), which mediate viral entry (Xu et al., 2021).

Clinical trials of the immunomodulatory and clinical effects of another RIPK1 inhibitor, SAR443122, in patients with severe COVID-19 are currently in progress. Interestingly, these studies conducted in LOs, show the applicability of these organoids as suitable models to dissect the pathological mechanisms underlying infectious diseases. Moreover, LOs can be used for rapid screening of the infectivity of emerging human airway pathogens and to test disease-specific therapeutics. Using LO models to test the safety and efficacy of potential drugs instead of time-consuming and costly clinical trials can greatly contribute to faster and cheaper development of new drugs. Indeed, an LO model has been used to test drugs for SARS-CoV-2 infection (Ebisudani et al., 2021). In this model, infected LOs were treated with physiologically relevant concentrations of lopinavir, nelfinavir, and remdesivir (Ebisudani et al., 2021). Lopinavir and nelfinavir are protein inhibitors commonly used in combination with other drugs for the treatment of HIV-1 infection in adults, adolescents and children (Croxtall and Perry, 2010). Remdesivir is an adenosine nucleotide analogue with broad-spectrum activity against viruses from various families (Lamb, 2020). Although lopinavir and nelfinavir significantly decreased the viral titer, SARS-CoV-2 viral replication was not halted. Interestingly, remdesivir inhibited the replication of the virus in infected organoids (Ebisudani et al., 2021). These results correlate with clinical data of patients infected by SARS-CoV-2 (Beigel et al., 2020; Grein et al., 2020; Ebisudani et al., 2021) and thus, suggest that LOs represent appropriate models for the study of infectious lung diseases.

To conclude, organoids have been already used successfully as models of human disease caused by infections with viruses such SARS-CoV-2 and RSV. These models represent effective and relatively cheap tools for preclinical and clinical trials of potential treatment strategies. Furthermore, LO models of human lung tissue and infectious disease offer possibilities for comparison with, and validation of, serological data from the patients in the clinic.

### 4.3 Lung Organoids in Cancer Research

Compared to conventional 2D monolayers and suspension cell cultures, LOs used in cancer research provide a valuable opportunity to develop physiologically, genetically, and histologically relevant tumor models. LOs can mimic some of the high cellular organization, tumor microenvironment, and cell interactions of *in vivo* tumor tissue. As such, LOs have been used in various contexts from carcinogenesis, to personalized medicine and drug development (Gunti et al., 2021).

Lung cancer organoids (tumoroids) precisely reflect the histological features of primary lung tumors and maintain their genomic abnormalities during long-term expansion *in vitro*. Recognizing that these models represent a valuable resource, Kim et al. (2019) created a biobank of 80 lung tumoroids from the five most frequent subtypes of lung cancers, and five normal bronchial organoids as controls, which replicate the unique histological features of the primary tissues. Such biobanks serve as a beneficial platform for drug testing and pre-clinical research to advance personalized medicine approaches.

Similar to LOs derived from primary lung progenitors, organoids established from lung cancer cell lines or primary tumor cells contain only a few cell types and so fail to completely replicate the tumor *in vitro* (Fiorini et al., 2020). Nevertheless, these systems have been used successfully in drug sensitivity studies. In one such study, high-throughput screening of anticancer agents on liver organoids indicated that patient-derived organoids can mimic a patient’s response to a particular therapy. Thus, these types of organoids could potentially be used for the development of personalized medicine approaches or as part of clinical trials (Vlachogiannis et al., 2018). Indeed, others have shown that cancer-cell-derived LOs can serve as a tool for pre-treatment drug screening and personalized medicine. Hai et al. (2020) developed genetically modified mouse LOs to mimic human lung squamous cell carcinoma (LSCC) and used this model to demonstrate that treatment with the WEE1 inhibitor (AZD1775) and the adjuvant PD-1 inhibitor enhanced T-cell anti-tumour activity. This demonstrates the importance of drug efficacy screening for identifying more efficient therapeutic drug combinations for evaluation in future clinical trials (Hai et al., 2020). Shi et al. (2020) published a protocol for *in vitro* generation of non-small cell lung cancer (NSCLC) organoids that mimic the histological attributes of the original tumor tissue. Moreover, these NSCLC organoids retained the molecular profile of matching tumor tissue long-term, even after multiple passages (>3 months, >10 passages). Next, they and others showed that NSCLC organoids and PDX models from the same parental tissue exhibit similar drug responses and proposed that organoid cancer models are a valid pre-clinical model to explore novel therapeutic options for NSCLC disease (Hai et al., 2020; Li et al., 2020; Shi et al., 2020; Wang et al., 2020b). Li et al. (2020) established organoid models of lung adenocarcinoma (LADC), the most common subtype of NSCLC. They also showed that the *in vitro* generated organoids preserve the key tumor features and therefore, can be used as a valuable pre-clinical tool. Using LADC organoids derived from 12 cancer lines they performed high-throughput drug response screening and showed that the sensitivity to a particular drug was consistent throughout individual passages and also that the response to the drug varied among different organoid lines (Li et al., 2020). Moreover, they discovered unpredicted drug sensitivity regardless of genetic markers. For example, the ACI-3_O line and SOL-4_O line responded gefitinib despite the lack of EGFR mutations. Again, their conclusions highlight the benefits of drug response screening on organoids (Li et al., 2020). Kim et al. (2019) used LOs generated from patient-derived cells to dissect the genetic and phenotypic basis of heterogeneous responses to anticancer therapy. They demonstrated that variability in drug responses correlated with particular genomic mutations and persisted during multiple organoid passages. For example, organoids with a *BRCA2* gene mutation showed increased sensitivity to the PARP inhibitor, Olaparib (Kim et al., 2019). Moreover, PDX derived from these organoids also responded to Olaparib treatment. On the other hand, although two organoids derived from different tissues expressed the same EGFR mutation, their responses to erlotinib and crizotinib differed due to secondary mutations. Overall, these studies highlight the critical importance of the LO biobank mentioned above for anti-tumor drug screening to predict individual patient drug responses (Kim et al., 2019).

The results mentioned above demonstrate that organoids are suitable for high-throughput screening while providing the advantages of 3D cancer models which, combined with clinical evaluation of specific genetic alterations, can serve as a tool for personalized therapy. Indeed, another recent study by Hu et al. (2021) proved that LOs successfully mimic original tumor tissue features and preserve them after *in vitro* cultivation. Moreover, they developed an integrated superhydrophobic microwell array chip (InSMAR-chip) suitable for high-throughput testing. With this set-up, they analyzed drugs administered during patient treatment and obtained results showing 100% accuracy and specificity in 10 of 21 samples. The remaining 11 on-chip samples could not be compared to relative patient responses due to differences in subsequent treatment. Nevertheless, this study showed a strong correlation of the drug response with genetic mutation and clinical outcomes and therefore, confirmed this as a promising approach for predicting the most effective drugs for individual patients (Hu et al., 2021).

As a result of such successes, LOs derived from patients with various lung pathologies have now entered clinical trials to establish their efficacy in predicting individual patients’ responses to a specific therapy (Table 2).

**TABLE 2:** Clinical trials in lung organoids: List of the clinical trials using 3D LOs as *in vitro* models.

Disease	Model	Source of the cells	Purpose of the study	ClinicalTrials.gov Identifier
Lung cancer	Spheroids	Lung tumor biopsies	Characterization of the consistency and accuracy of the organoids derived from patient lung biopsies to predict clinical response to the chemotherapy	NCT03979170
	Patient-derived LOs			
	3D model OncoCilAir™ (OncoTheis)			
Lung cancer	Patient-derived normal and cancer LOs	Biopsies from endobronchial tumors or lymph nodes	Biobanking of normal and primary lung cancer organoids. Analysis of microvesicles secreted by lung cancer cells in organoid-derived culture supernatants and patient blood samples. Comparison of the response to the drugs in normal and cancer LOs	NCT05092009
	Blood samples			
Lung cancer	Patient-derived organoids	Tumor tissue biopsies	Comparison of the xenografts with donor tissue. Testing novel anti-cancer treatment. Developing assays to predict tumor response to the drug	NCT04859166
	Xenografts			
CF	Organoids derived from the tissue of patients with the R334W-CFTR mutation	Rectal biopsies	Study of the response of organoids to CFTR modulators, which will be compared to the patients’ response to the same drug in the next study	NCT04254705
Lung cancer	Patient-derived LOs	Lung tumor biopsies	Biobanking of organoids derived from stage I–IV lung cancer patients	NCT03655015
Lung cancer	Patient-derived LOs	Non-small cell lung cancer patient biopsies	Use of organoids for the drug sensitivity testing and comparison with clinical treatment data	NCT03453307
COPD and IPF	Patient-derived LOs	Lung tissue biopsies from patients with emphysema or pulmonary fibrosis	Characterization of the stem cell niche in different tissues (healthy, emphysematous and fibrotic pulmonary tissue). Further use of the organoids for drug screening and personalized medicine	NCT02705144
Lung cancer	Patient-derived LOs	Non-small cell lung cancer patient biopsies	Testing of different drugs *in vitro* using organoids. Evaluation of the responders to Osimertinib and screening of alternative therapies for non-responders *in vitro*	NCT05136014
Lung cancer	Patient-derived LOs	Tumor biopsies	Use of organoids and a microfluidic system as an innovative model of the tumor microenvironment and HUVECS or endothelial cells as a model of tumor vascularization, to create a tool for personalized medicine	NCT04826913
	Microfluidic system	Blood samples		
Lung cancer	Patient-derived organoids from lung tumors or other solid tumors	Lung cancer tissue or solid tumor biopsies	Co-cultivation of organoids with lymphocytes to screen for tumor-responsive T-cells, which will be further expanded and used as immunotherapy for the patient	NCT03778814
	TILs or/and peripheral T-cells			
CF	Patient-derived organoids	Not specified	Use of an *ex vivo* organoid model to establish the correlation between the clinical response of CF patients to Vx-770 (Ivacaftor)	NCT03390985

Used abbreviations: COPD, chronic obstructive pulmonary disease; CF, cystic fibrosis; IPF, idiopathic pulmonary fibrosis; LOs, lung organoids; 3D, three-dimensional; CFTR, cystic fibrosis transmembrane conductance regulator.

These studies of high-throughput screening have highlighted the potential of cancer organoids as a promising tool for personalized treatment. Using such progressive methods, organoids can be generated for each individual patient and used to screen drug responses based on the initial clinical tests of specific genetic mutations and gene expression profiles. This approach holds great potential for achieving better clinical outcomes and prolonged survival of patients. High-throughput testing also offers a great advantage for screening samples currently available in biobanks. Furthermore, as more patient-derived samples are contributed, the collection of unique genetic alterations and their specific combinations will expand. Thus, we expect that this approach will provide a standardized tool for rapid development and testing of new anti-tumor treatments.

### 5 Lung Organoids: Future Prospects and Remaining Challenges

So far, we have discussed the great progress made using primary lung progenitor cell-derived and iPSC-derived LOs in modeling human diseases such as CF, IPF, lung cancer, and SARS-CoV-2 infection. However, certain challenges still need to be addressed to improve the relevance of these models to *in vivo* conditions. Several approaches have been developed to further enhance the physiological resemblance of *in vitro* 3D models to lung tissue *in vivo*. One is based on the cultivation of lung epithelial cells at an air-liquid interface, where the basolateral side of the epithelial cells is immersed in culture medium while the apical side is exposed to humidified air. This approach was used by Mas et al. (2016) to develop OncoCilAir™ tumor-stroma airway model by co-culture of lung adenocarcinoma cells, human primary bronchial cells, and lung fibroblasts. This model provides an alternative means of testing drug efficacy and toxicity that could replace animal models by overcoming the disadvantages of differences in rodent cancer physiology and genetic features. Specifically, the major drawback of animal lung models is the diversity in progression of lung disease and response to therapy (Mas et al., 2016).

The newly developed cultures that incorporate an extracellular scaffold represent a further advance that enables standardized long-term cultivation conditions and reproducibility (Zscheppang et al., 2018). Moreover, this approach facilitates exploration of the interaction between the ECM and lung cells in the context of aberrant repair or regeneration. The scaffolds are generated by decellularization of healthy human lung tissue or tissue derived from patients with conditions such as IPF. These models will contribute greatly to our understanding of the pathogenesis of lung disease and evaluation of the role of ECM in the onset and course of these pathologies (Zscheppang et al., 2018).

While 3D models have many advantages compared to conventional 2D models, many still fail to fully represent even a small fraction of the dynamic features of human lungs, such as the processes of nutrient and gas exchange, the mechanical forces created by breathing movements, and dynamic flow conditions. The tumor-on-a-chip model however, was designed to overcome these limitations (Barros et al., 2021). These microfluidic models of lung tumors are often derived from established lung cancer cell lines, which form a spheroid in the microchannel, adding a further 3D aspect to these studies (Mehta et al., 2022). Microfluidic devices have been used to study drug resistance, the efficiency of photodynamic therapy, the influence of mechanical forces in the lungs on tumor progression and drug resistance, and characterization of cell-cell communication in the tumor microenvironment (Del Piccolo et al., 2021). For example, Xu et al. (2013) developed a microfluidic device to study drug sensitivity of a co-cultured human non-small cell lung cancer cell line (SPCA-1) and stromal cells. They also established a culture of fresh lung cancer tissues from eight patients in their microchip device, in order to identify personalized medical treatments. This approach accomplished replication of the actual condition of the solid tumor *in vivo*. Microfluidic devices provide an easy way to administer single or combined anti-cancer treatments in high-throughput assays, which is beneficial for the development of personalized medicine. Further advantages are that these devices require only small amounts of samples and reagents, short assay time and high sensitivity. Microfluidic devices can also be used with various tissue set-ups, such as cell line monolayers or co-culture of cancer cell lines and stromal cells, to more accurately recapitulate tumor tissue *in vivo*, or even primary fresh tissue. This approach revealed significant differences in treatment sensitivity, with the poorest detected in fresh tissue (Xu et al., 2013). Thus, further studies are warranted to develop methods for cultivation of patient tumor tissue *in vitro* while closely maintaining the physiological features of the human body that can be used to exploit the potential of organoids in the development of personalized therapy.

Sepsis and septic shock greatly contribute to the mortality of human population (Rudd et al., 2020). Therefore, the development of an *in vitro* model of sepsis represents another prospect for the LO approach for defining the exact pathogenesis of the disease and identifying potential therapeutic strategies and new biomarkers. However, an *in vitro* model of sepsis-induced lung injury based on LO technology may require a complex set-up due to the number of different cell types and their specific effects in septic reactions. For instance, hypoxemia, which is an important factor in the pathophysiology of ALI/ARDS, is caused by neutrophil entrapment in the pulmonary microvasculature (Park et al., 2019). Therefore, to create a relevant *in vitro* model of sepsis might warrant culture under hypoxic conditions. Nevertheless, similar to the situation for infectious diseases, a great advantage of LOs as a model of human sepsis is in the possibility of comparison with, and validation of, serological data from patients in the clinic.

The use of iPSCs to derive LOs has rapidly advanced the field in recent years; however, these models are not yet perfect. LOs developed from iPSCs resemble fetal tissues rather than adult lungs and, therefore, transplantation into a living organism may be required to fully recapitulate the adult lung phenotype (Dye et al., 2015). This engraftment procedure has been successfully achieved using humanized/immunodeficient mouse recipients to develop mature intestinal (Cortez et al., 2018; Poling et al., 2018; Boyle et al., 2021), brain (Dong et al., 2021), and kidney (van den Berg et al., 2018) organoids, but it is not so commonly practiced with LOs. An early study by Dye et al. (2016) demonstrated that human ESC-derived LOs can be maintained for up to 15 weeks after engraftment into the epididymal fat pad of male NOD-scid IL2Rgnull mice. After the engraftment, LOs improved their cellular differentiation of secretory lineages and formed airway-like structures that were similar to adult human lungs, including vasculature and smooth muscles. However, a bioartificial microporous poly (lactide-co-glycolide) (PLG) scaffold niche was necessary for proper engraftment (Dye et al., 2016), which requires advanced techniques for its fabrication. Nevertheless, alternative approaches such as decellularized lung scaffolds are also available to provide relevant physical and environments for the generation of organized lung structures (Gilpin et al., 2014).

**FIGURE 5:**
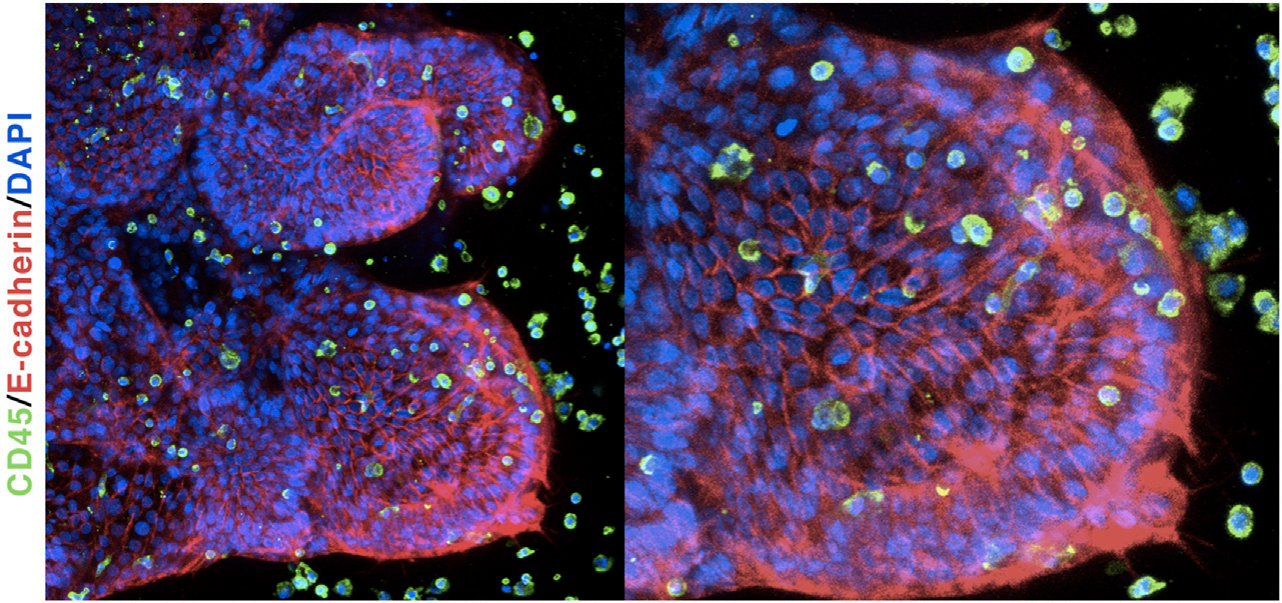
Lung organoids co-cultured with primary monocytes: Immunofluorescent labeling of human LOs shows tissue polarity and recruitment of human monocytes.

A notable drawback of iPSC-derived LOs is the lack of a pulmonary immune cell populations. The human lung comprises both tissue-resident immune cells (such as pulmonary macrophages) as well as infiltrating immune cells, such as neutrophils, monocytes, and T-cells, which are not found in LOs. This immune cell-deprived scenario renders such a model unsuitable for studying diseases in which immune cells have a major role—including microbial infections and inflammation. However, recent studies have demonstrated that immune cells can be co-cultured with organoids to generate a more physiologically relevant model. Our own findings show that iPSC-derived LOs, although deprived of immune cell populations, can respond to microbial ligands and are able to recruit human primary monocytes *in vitro* (Jose et al., 2020). Interestingly, co-cultured monocytes interacted closely with the organoid tissue and significantly changed their phenotype (Figure 5). Similarly, another study showed that RSV-infected airway organoids were able to recruit and interact with human neutrophils (Sachs et al., 2019). Taken together, these results suggest that co-culturing LOs with primary immune cells will allow a better dissection of the interactions of immune cells with human tissues and thus, contribute to a better understanding of complex pathologies.

As already mentioned, the local microbiota plays a crucial role in the pathology and homeostasis of the lung tissue (Paolicelli et al., 2019; Barcik et al., 2020). Although many studies have focused on the role of the microbiota in the gastrointestinal tract using human intestinal organoids (Min et al., 2020), few studies using LOs have been reported. Therefore, future research should focus on dissection of the cell-cell interaction of the microbiota with lung tissue and the molecular mechanisms by which the microbiota influences the development, pathology, and homeostasis of human lung.

## 6 Discussion

Using 3D organoids as a tool to understand lung development and function holds great potential for generating important insights not only into genetic lung disorders, but also into diseases such as COPD, CF, and lung infections. The generation of LOs from human PSCs is crucial for overcoming the obstacles to *in vitro* studies of human cells, which for many years have relied on the scant availability of post-mortem tissues. LOs can bridge the knowledge gap between lung disease pathways identified in rodent models and therapeutic possibilities in their human counterparts. The end of our long-term dependence on rodent models for genetic manipulation studies may also be heralded by the emergence of novel genome editing techniques such as CRISPR, which could be used to generate genetic models of pulmonary disease using organoids. This approach would facilitate mechanistic studies in a physiological setting that closely resembles the human respiratory system without the need for presumably costly and time-consuming animal research. The recent advances in the use of iPSC-derived LO in clinical trials are also promising in terms of drug screening for personalized treatment or transplantation of healthy tissues, especially allogenic tissues, in patients, and hints at a future that includes “organoid-based treatment” of pulmonary diseases.

In summary, we believe that organoids represent the future of lung disease modeling as they allow long-term cultivation of human lung tissue, maintaining many of its phenotypical, and functional characteristics. LOs represent one of the best tools for translational research, due to the comparatively inexpensive and increasingly biologically-relevant methodology. The past 10 years have seen an increasing number of studies leveraging organoids to shed light on previously unexplained pathologies of diseases such as IPF (Chen et al., 2017; Kim et al., 2021; Suezawa et al., 2021), CF (Dekkers et al., 2013; Firth et al., 2015; Kim et al., 2021), and lung cancer (Hai et al., 2020; Li et al., 2020; Shi et al., 2020; Wang et al., 2020b; Gunti et al., 2021; Hu et al., 2021). Harnessing the potential that LOs have to offer will promote the design of new treatments and diagnostic methodologies and thus, finally improve the quality of life of people affected by various pulmonary diseases.
